# The concentration of oxygen, lactate and glucose in the central veins, right heart, and pulmonary artery: a study in patients with pulmonary hypertension

**DOI:** 10.1186/cc5739

**Published:** 2007-04-11

**Authors:** Guillermo Gutierrez, Anthony Venbrux, Elizabeth Ignacio, Jonathan Reiner, Lakhmir Chawla, Anish Desai

**Affiliations:** 1 Division of Pulmonary and Critical Care Medicine, Department of Medicine, The George Washington University Medical Center, Pennsylvania Avenue, NW Washington, District of Columbia, 20037, USA; 2 Department of Radiology, The George Washington University Medical Center, Pennsylvania Avenue, NW Washington, District of Columbia, 20037, USA; 3 Division of Cardiology, Department of Medicine, The George Washington University Medical Center, Pennsylvania Avenue, NW Washington, District of Columbia, 20037, USA; 4 Department of Anesthesiology and Critical Care Medicine, The George Washington University Medical Center, Pennsylvania Avenue, NW Washington, District of Columbia, 20037, USA

## Abstract

**Introduction:**

Decreases in oxygen saturation (SO_2_) and lactate concentration [Lac] from superior vena cava (SVC) to pulmonary artery have been reported. These gradients (ΔSO_2 _and Δ[Lac]) are probably created by diluting SVC blood with blood of lower SO_2 _and [Lac]. We tested the hypothesis that ΔSO_2 _and Δ[Lac] result from mixing SVC and inferior vena cava (IVC) blood streams.

**Methods:**

This was a prospective, sequential, observational study of hemodynamically stable individuals with pulmonary artery hypertension (*n *= 9) who were about to undergo right heart catheterization. Catheters were advanced under fluoroscopic guidance into the IVC, SVC, right atrium, right ventricle, and pulmonary artery. Samples were obtained at each site and analyzed for SO_2_, [Lac], and glucose concentration ([Glu]). Analysis of variance with Tukey HSD test was used to compare metabolite concentrations at each site.

**Results:**

There were no differences in SO_2 _or [Lac] between IVC and SVC, both being greater than their respective pulmonary artery measurements (*P *< 0.01 for SO_2 _and *P *< 0.05 for [Lac]). SO_2 _and [Lac] in right atrium, right ventricle, and pulmonary artery were similar. ΔSO_2 _was 4.4 ± 1.4% (mean ± standard deviation) and Δ[Lac] was 0.16 ± 0.11 mmol/l (both > 0; *P *< 0.001). Δ[Glu] was -0.19 ± 0.31 mmol/l, which was not significantly different from zero, with SVC [Glu] being less than IVC [Glu].

**Conclusion:**

Mixing of SVC with IVC blood does not account for the development of ΔSO_2 _and Δ[Lac] in hemodynamically stable individuals with pulmonary artery hypertension. An alternate mechanism is mixing with coronary sinus blood, implying that ΔSO_2 _and Δ[Lac] may reflect changes in coronary sinus SO_2 _and [Lac] in this patient population.

## Introduction

Blood oxygen saturation (SO_2_) in the superior vena cava (SVC) is approximately 2% to 5% higher than that in the pulmonary artery [1,2]. This SVC-pulmonary artery gradient in SO_2 _varies considerably among individuals, or even within the same person when it is measured at different times [3]. Declines in blood lactate concentration ([Lac]) from right atrium to pulmonary artery (Δ[Lac]) have also been reported [4]. The SO_2 _and [Lac] gradients (ΔSO_2 _and Δ[Lac]) probably develop as SVC blood mixes with blood from the inferior vena cava (IVC) or from the heart's venous drainage, comprised of blood emanating from the coronary sinus and Thebesian veins; alternatively (and more likely), blood from both sources mixes at varying proportions [5].

Monitoring ΔSO_2 _and Δ[Lac] may be of little clinical interest if these gradients are produced exclusively by mixing of SVC and IVC blood streams. On the other hand, if ΔSO_2 _and Δ[Lac] result from mixing of SVC with coronary venous blood, either in part or in whole, then it is possible for these gradients to reflect alterations in myocardial oxidative metabolism [4,6]. The heart is the most aerobic of organs, normally deriving its energy from the oxidation of free fatty acids and lactate, and coronary venous blood normally has the lowest SO_2 _of any venous blood [7]. Moreover, myocardial lactate oxidation accounts for 10% to 20% of total myocardial aerobic energy production, and coronary venous [Lac] is substantially lower than that of other venous effluents [8].

To test the hypothesis that ΔSO_2 _and Δ[Lac] result exclusively from mixing of SVC and IVC blood streams, we measured concentrations of oxygen and [Lac] in the central veins, the right heart chambers, and the pulmonary artery of hemodynamically stable individuals who were about to undergo right heart catheterization. Additionally, we measured the glucose concentration ([Glu]) in the aforementioned sites, because this substrate is also known to play an important role in myocardial energy metabolism.

## Materials and methods

This was a prospective, sequential observational study conducted in persons of either sex admitted to The George Washington University Hospital with a diagnosis of pulmonary artery hypertension who were scheduled to undergo right heart catheterization. The institutional review board approved the study with the exclusion of drawing arterial blood samples. All patients underwent cardiac catheterization in order to evaluate cardiac function and pulmonary artery pressures, and were not healthy volunteers. Written informed consent was obtained from each patient.

Nine individuals who were older than 18 years, of either sex, were enrolled sequentially in the study. All patients were ambulatory. Patients were sedated before the procedure with 4 to 8 mg midazolam intravenously. Electrocardiograph leads were monitored continuously and arterial blood pressure was measured in the right arm at 1 min intervals using an automated inflatable blood pressure measuring device. Supplemental oxygen by mask was given to maintain arterial SO_2_, measured by pulse oximetry, above 98% at all times during the procedure. An 8 Fr venous sheath was placed in the right femoral vein and a 7 Fr Van Aman pigtail catheter (Cook, Bloomington, IN, USA) was inserted under sterile technique and guided under fluoroscopy into the IVC just above the diaphragm (IVC site). It was then advanced successively into the SVC, approximately 5 cm above the right atrium (SVC site), the right atrium (right atrium site), the right ventricle (right ventricle site), and pulmonary artery (pulmonary artery site). A small amount of non-ionic contrast media was injected with the catheter in the right atrium to rule out the presence of a patent foramen ovale or septal defects. In one individual the catheter was inserted through the right jugular vein and proper positioning at sampling each site was also confirmed fluoroscopically. Measurement of hydrostatic blood pressure at each site was followed by the drawing of 1.5 ml blood aliquots, with the first 2 ml of blood drawn from the catheter discarded to prevent contamination with flushing fluid. The Van Aman catheter was removed and exchanged for a 7.5 Fr pulmonary artery catheter (Swan-Ganz standard thermodilution pulmonary artery catheter; Edwards Life Sciences, Irvine, CA, USA) and measurements were taken of cardiac output in triplicate using the thermodilution method and pulmonary artery occlusion pressure. Cardiac index was computed by dividing cardiac output by the patient's body surface area.

Blood samples were immediately placed in ice and promptly analyzed in triplicate [9] for SO_2 _saturation (IL682 CO-Oximeter; Instrumentation Laboratories, Lexington, MA, USA), and [Lac] and [Glu] (YSI 2300 STAT Plus Lactate/Glucose Instrument; YSI Company, Yellow Springs, OH, USA). The YSI 2300 STAT Plus measures [Lac] and [Glu] in whole blood and has been used in studies of blood [Lac] in critically ill individuals [10.11]. The accuracy of whole blood lactate measurements, as compared with those in plasma, was previously established [12]. The reported precision of blood lactate measurements [13] with the YSI 2300 STAT Plus is 0.06 mmol/l for lactate values below 2.5 mmol/l. In the present study, we found the precision of the three repeated measurements to be 0.86% for SO_2_, 0.09 mmol/l for [Lac], and 0.21 mmol/L for [Glu].

### Statistical analysis

Analysis of variance for repeated measures was used to compare mean SO_2_, [Lac], and [Glu] at each sampling site. The Tukey HSD test [14] was performed for multiple comparisons among sampling sites whenever the F ratio was significant. The gradient Δ in the various parameters is defined as the difference between SVC and pulmonary artery. Unless stated otherwise, data are expressed as mean ± standard deviation, with *P *< 0.05 denoting a statistically significant difference.

## Results

**Figure 1:**
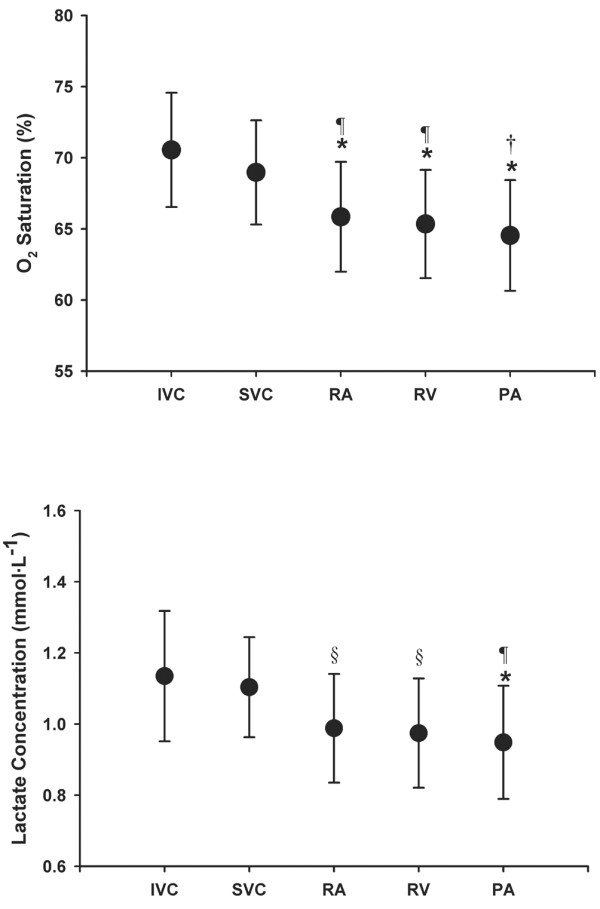
Oxygen saturation and lactate concentration at the various sampling sites. Nine patients were included. Values are expressed as mean ± standard error. *P < 0.01, ^§^*P *< 0.05 comparing right atrium (RA), right ventricle (RV) and pulmonary artery (PA) versus inferior vena cava (IVC). ^†^*P *< 0.01, ^¶^*P *< 0.05 comparing RA, RV and PA versus superior vena cava (SVC).

Table 1 shows mean hydrostatic blood pressures measured at each sampling site as well as pulmonary artery occlusion pressure, mean arterial pressure, and cardiac index. Table 2 shows individual SO_2 _and [Lac] measured at each sampling site, and Figure 1 shows graphs of mean ± standard error values for SO_2 _and [Lac]. There were no differences in SO_2 _between IVC and SVC. SO_2 _levels at the IVC and SVC were greater than that at the pulmonary artery (*P *< 0.01) and were greater than SO_2 _at the right atrium and right ventricle sites (*P *< 0.01 for IVC and *P *< 0.05 for SVC). There were no differences in SO_2 _among right atrium, right ventricle, and pulmonary artery sites. ΔSO_2 _was 4.4 ± 1.4%, which was significantly different from zero (*P *< 0.001).

**Table 1:** Hydrostatic pressures and cardiac index

Parameter	Value
IVC (mmHg)	18.1 ± 8.2
SVC (mmHg)	13.0 ± 7.4
RA (mmHg)	14.0 ± 11.0
RV (mmHg)	20.5 ± 7.6
Pulmonary artery (mmHg)	38.4 ± 14.1
PAOP (mmHg)	14.6 ± 11.6
MAP (mmHg)	93.9 ± 11.9
CI (l/min per m^2^)	2.6 ± 0.6

Nine patients were included. Values are expressed as mean ± standard deviation. CI, cardiac index; IVC, inferior vena cava; MAP, mean arterial pressure; PAOP, pulmonary artery occlusion pressure; RA, right atrium; RV, right ventricle; SVC, superior vena cava.

**Table 2:** Individual measurements of SO_2 _and [Lac], and their gradients, obtained by sampling different sites during right heart catheterization

Patient number	IVC	SVC	RA	RV	PA	ΔSO_2 _or Δ[Lac]
SO_2 _(%)						
1	80.9	76.5	75.6	72.5	71.9	ΔSO_2 _= 4.7
2	77.5	69.1	65.6	66.5	64.5	ΔSO_2 _= 4.6
3	72.9	69.2	67.7	67.5	66.3	ΔSO_2 _= 2.9
4	68.2	67.4	63.1	62.8	61.5	ΔSO_2 _= 5.9
5	61.9	59.2	52.6	51.9	52.6	ΔSO_2 _= 6.6
6	78.7	84.5	79.2	80.0	79.7	ΔSO_2 _= 4.7
7	84.7	81.5	80.6	79.2	79.7	ΔSO_2 _= 1.8
8	59.8	55.9	55.2	55.9	52.7	ΔSO_2 _= 3.2
9	50.3	57.4	53.1	51.8	51.9	ΔSO_2 _= 5.5
Mean	70.5	69.0	65.8*^†^	65.3*^†^	64.5*^‡^	ΔSO_2 _= 4.4
SD	11.4	10.4	10.9	10.8	11.0	ΔSO_2 _= 1.6
[Lac] (mmol/l)						
1	0.43	0.55	0.41	0.40	0.38	Δ[Lac] = 0.17
2	2.17	1.98	1.91	1.95	1.93	Δ[Lac] = 0.05
3	1.07	1.00	0.89	0.87	0.90	Δ[Lac] = 0.10
4	1.39	1.24	1.25	0.97	0.89	Δ[Lac] = 0.34
5	1.53	1.25	1.18	1.20	1.24	Δ[Lac] = 0.01
6	0.63	0.86	0.62	0.66	0.63	Δ[Lac] = 0.23
7	1.09	0.86	0.79	0.79	0.63	Δ[Lac] = 0.23
8	1.04	1.21	0.98	1.08	1.09	Δ[Lac] = 0.12
9	0.86	0.98	0.86	0.86	0.85	Δ[Lac] = 0.14
Mean	1.13	1.10	0.99^§^	0.97^§^	0.95*^†^	Δ[Lac] = 0.16
SD	0.52	0.40	0.43	0.43	0.45	Δ[Lac] = 0.10

Nine patients were included. * *P *< 0.01, ^§^*P *< 0.05 compared with IVC. ^‡^*P *< 0.01, ^†^*P *< 0.05 compared with SVC. CI, cardiac index; IVC, inferior vena cava; [Lac], lactose concentration; MAP, mean arterial pressure; PAOP, pulmonary artery occlusion pressure; RA, right atrium; RV, right ventricle; So2, oxygen saturation; SVC, superior vena cava.

There were no differences in [Lac] between the IVC and SVC sites. IVC [Lac] and SVC [Lac] were greater than pulmonary arterial [Lac] (*P *< 0.01 for IVC and *P *< 0.05 for SVC). IVC [Lac] was also greater than right atrial and right ventricular [Lac]. There were no differences in [Lac] among right atrium, right ventricle and pulmonary artery sites. Δ[Lac] was 0.16 ± 0.11 mmol/l, which was significantly different from zero (*P *< 0.001).

**Figure 2:**
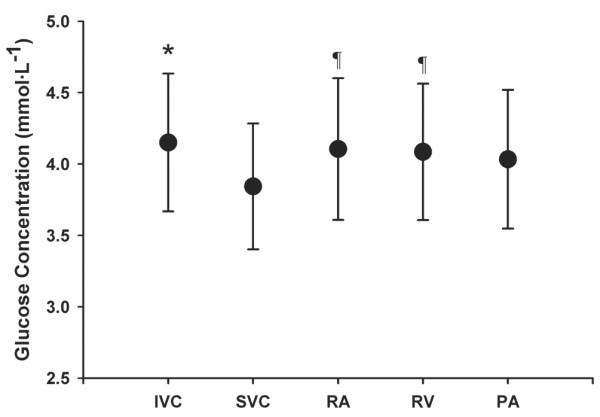
Glucose concentration at the various sampling sites. Nine patients were included. Values are expressed as mean ± standard error. *P < 0.01 comparing inferior vena cava (IVC) versus superior vena cava (SVC). ^¶^*P *< 0.05 comparing right atrium (RA) and right ventricle (RV) versus SVC. PA, pulmonary artery.

Figure 2 shows the [Glu] values for each sampling site. [Glu] at the SVC was significantly lower than that at the IVC, right atrium, and right ventricle sites (*P *< 0.01, *P *< 0.05, and *P *< 0.05, respectively). There were no differences in [Glu] among the IVC, right atrium, right ventricle, and pulmonary artery sites. Δ[Glu] was -0.19 ± 0.31 mmol/l, which was not significantly different from zero.

## Discussion

The aim of the present study was to test the hypothesis that the mechanism responsible for the development of SO_2 _and [Lac] gradients from SVC to pulmonary artery is mixing of SVC with IVC blood. To that end, we measured the steady state concentration of oxygen and [Lac] in the central veins, the right heart chambers, and the pulmonary artery in hemodynamically stable individuals who were suspected of having elevated pulmonary artery pressures.

Several studies [1-3,15-26] have compared SO_2 _in SVC with that in the pulmonary artery. The majority of these studies found decreases in SO_2 _as blood travels from SVC to pulmonary artery. The average ΔSO_2 _of 4.4% found in the present study agrees with mean values of 3% to 5% reported by others. According to our results, however, mixing of SVC with IVC blood cannot account for the development of ΔSO_2 _and Δ[Lac], as noted in this patient population. The average concentrations of oxygen and lactate in SVC and IVC blood were indistinguishable from each other. Moreover, SO_2 _and [Lac] in the SVC and IVC were both greater than the respective pulmonary artery values. Therefore, it would be physically impossible for the mixing of SVC and IVC blood streams to produce pulmonary artery blood of lesser SO_2 _and [Lac].

The numerical average of IVC and SVC SO_2 _(mean SO_2 _= [IVC SO_2 _+ SVC SO_2_]/2) provides a first order estimate of right atrial SO_2_. Assuming no input whatever from other venous sources, such as coronary sinus, the estimate for right atrial SO_2 _computed in this manner should equal pulmonary arterial SO_2_. Table 3 shows differences between computed mean SO_2 _and pulmonary arterial SO_2 _reported in published studies measuring SO_2 _in the central veins, the right heart, and pulmonary artery in humans [27-31]. With the exception of a subset of eight patients who were 'not in shock', reported by Lee and coworkers [31], all studies find that mean SO_2 _was greater than pulmonary arterial SO_2_. The combined average difference for the group is 1.82 ± 0.78%, a value significantly different from zero (*P *< 0.05). These data also fail to support the hypothesis of mixing SVC with IVC blood as the sole mechanism for ΔSO_2 _in hemodynamically stable individuals. Given that two-thirds of the systemic venous return in adults is via the IVC [32], the magnitude of the difference between mean SO_2 _and pulmonary arterial SO_2 _would have been even greater if more weight had been placed on IVC SO_2 _in the computation of mean SO_2_.

**Table 3:** Differences between the calculated mean SO_2 _and pulmonary artery SO_2_

Reference	Mean SO_2 _– pulmonary arterial SO_2_
Barrat-Boyes and Wood [27]	1.5%
Gasul and coworkers [28]	2.0%
Gutgesell and Williams [29]	1.5%
Kjellberg and coworkers [30]	2.5%
Lee and coworkers (not in shock) [31]	-1.2%
Lee and coworkers (shock) [31]	1.2%
Present study	5.2%

Shown are differences in SO_2 _(%) between pulmonary artery SO_2 _and calculated SO_2_, where mean SO_2 _= (IVC SO_2 _+ SVC SO_2_)/2. IVC, inferior vena cava; SO_2_, oxygen saturation; SVC, superior vena cava.

Few studies have reported on the distribution of SO_2 _in the central veins and right heart in shock states. Lee and coworkers [31] noted that IVC SO_2 _and SVC SO_2 _were 49.1% and 65.8%, respectively, in five patients with cardiogenic shock (cardiac index 1.7 l/min per m^2^). Pulmonary arterial SO_2 _was nearly equal to the computed mean SO_2_, indicating a predominant role for IVC SO_2 _in the formation of ΔSO_2_. No comparable studies in septic shock have been reported. Dahn and coworkers [33] measured hepatic venous SO_2 _in 15 septic patients and found a normal pulmonary arterial SO_2 _of 70.5% at a time when hepatic venous SO_2 _was 55.6%. Similar findings were reported by De Backer and colleagues [34], who measured hepatic venous SO_2 _in 42 septic patients and noted pulmonary arterial SO_2 _and hepatic venous SO_2 _to be 67.3% and 50.3%, respectively. Little insight can be gained from these data into the genesis of ΔSO_2 _in septic shock, because neither IVC SO_2 _nor SVC SO_2 _were measured in these studies.

Ours is the only study to report the distribution of [Lac] in the central vasculature, and only two other studies have compared lactate concentrations in SVC and pulmonary artery. Weil and coworkers [35] found no differences between SVC [Lac] and pulmonary arterial [Lac] in 12 patients. Conversely, we measured a Δ[Lac] of 0.2 mmol/l in 45 critically ill individuals in which blood samples were obtained from the proximal and distal ports of pulmonary artery catheters [4]. The present study corroborates our previous finding that a measurable [Lac] gradient exists between SVC and pulmonary artery. We also noted that pulmonary arterial [Lac] was lower than either SVC [Lac] or IVC [Lac], a finding that also refutes the idea of mixing SVC and IVC blood as the mechanism for development of Δ[Lac].

The finding of greater SO_2 _and [Lac] in IVC and SVC than in pulmonary artery indicates that further dilution of oxygen and [Lac] takes place as blood flows through the right heart chambers. Given the vigorous myocardial extraction of oxygen and lactate, venous concentrations of those chemical species are lowest in coronary venous blood, which includes blood emanating from coronary sinus and the Thebesian system. Therefore, it is possible that blood flowing from the coronary sinus and Thebesian veins exerted a small but measurable diluting effect on right atrial SO_2 _and right atrial [Lac]. We lacked direct samples of coronary venous blood and cannot prove this hypothesis conclusively from the data presented. Lending support this notion, however, are the observations that significant decreases in SO_2 _and [Lac] occurred mainly in the right atrium, which is the anatomical location of the coronary sinus (Figure 1).

We found that the distribution pattern for [Glu] differed from those of SO_2 _and [Lac]. SVC [Glu] was lower than IVC [Glu], reflecting the high rate of cerebral glucose uptake. In adult humans glucose represents the main, if not the sole, substrate of brain energy metabolism, with the brain utilizing approximately 25% of circulating blood glucose [36,37]. In contrast to the distributions noted for SO_2 _and [Lac], right atrial [Glu] was greater than SVC [Glu] but nearly equal to IVC [Glu]. This concentration distribution is that expected for a metabolite whose coronary sinus concentration approximates that of SVC blood, such as may be the case for glucose in fully aerobic conditions [7]. It remains to be seen whether the [Glu] pattern changes with myocardial hypoxia, as glucose becomes the preferred metabolic substrate of the heart and coronary sinus [Glu] declines in relation to IVC [Glu] [8].

The individuals studied had elevated pulmonary arterial pressures, and patients with pulmonary arterial hypertension frequently have right-sided valvular regurgitation, right ventricular dilatation, and right-to-left shunts through a patent foramen ovale. Angiography did not reveal patent foramina or septal defects in any of the patients included in this study. Given their moderate severity of pulmonary arterial hypertension, right ventricular dilatation and pulmonary and tricuspid regurgitation in this particular group of patients were likely to have been modest. On the other hand, it is conceivable that retrograde transvalvular flow through the pulmonary valve could have affected right ventricular and pulmonary arterial values. Samples were obtained sequentially, not simultaneously, and the possibility exists that temporal changes in concentration occurred in the different sampling sites as the catheter was advanced into the pulmonary artery. To avoid this possibility, care was taken to verify with fluoroscopy the position of the catheter at each sampling site and the blood sampling procedure was performed within a span of 5 min, with no changes noted in heart rate or blood pressure in any of the patients. Finally, Δ[Lac] and Δ[Glu] were small when compared with the precision of the measuring instrument. This raises an important question regarding the utility of single measurements of Δ[Lac] and Δ[Glu], a question that only can be answered by further clinical studies.

## Conclusion

The development of ΔSO_2 _and Δ[Lac] in the patient population studied cannot be explained by the mixing of SVC and IVC blood. The development of these gradients appears to require mixing with blood of lower SO_2 _and [Lac], most likely blood emanating from the coronary sinus and Thebesian veins. Because coronary venous blood SO_2 _and [Lac] vary according to the rates of oxygen and lactate utilization by the heart, this mechanism suggests a possible role for ΔSO_2 _and Δ[Lac] as markers of myocardial energy metabolism in hemodynamically stable individuals [6]. Further work remains to be done to establish the provenance of these gradients in other clinical conditions, including shock states [38].

## Key messages

• Blood SO_2 _and [Lac] gradients exist from SVC to pulmonary artery.

• These gradients were not produced by mixing of SVC blood with IVC blood in a population of patients with mild-to-moderate pulmonary hypertension.

• Decreases in SO_2 _and [Lac] were noted to be greatest in the right atrium, suggesting that mixing of SVC with coronary venous blood is the primary mechanism resulting in ΔSO_2 _and Δ[Lac].

• Coronary venous blood SO_2 _and [Lac] vary according to their rates of utilization by the heart, and so it may be possible for ΔSO_2 _and Δ[Lac] to serve as markers of myocardial energy metabolism in the patient population studied.

## Abbreviations

[Glu] = glucose concentration; IVC = inferior vena cava; [Lac] = lactate concentration; SO_2 _= oxygen saturation; SVC = superior vena cava.

## Competing interests

GG has served in the past as consultant to Hospira, Inc., a manufacturer of pulmonary artery catheters. Hospira Inc. was not involved in any aspect of the study. GG holds a patent on a method related to the subject matter of the study. None of the other authors declare any competing interests.

## Authors' contributions

GG conceived and designed the study, analyzed, interpreted the data and wrote and reviewed the manuscript. AV acquired data and reviewed the manuscript. EI designed the study, acquired data, and reviewed the manuscript. JR acquired data, and reviewed the manuscript. LC designed the study, and reviewed the manuscript. AD designed the study, acquired data, and wrote and reviewed the manuscript.
